# Time associations between U.S. birth rates and add-Ons to IVF practice between 2005–2016

**DOI:** 10.1186/s12958-021-00793-2

**Published:** 2021-07-13

**Authors:** Norbert Gleicher, Lyka Mochizuki, David H. Barad

**Affiliations:** 1 The Center for Human Reproduction, New York, NY 10021 USA; 2 Stem Cell Biology and Molecular Embryology Laboratory, The Rockefeller University, New York, NY 10065 USA; 3 Foundation for Reproductive Medicine, New York, NY 10021 USA; 4 Department of Obstetrics and Gynecology, Vienna University School of Medicine, 1009 Vienna, Austria

## Abstract

Until 2010, the National Assisted Reproductive Technology Surveillance System (NASS) report, published annually by the Center for Disease Control and Prevention (CDC), demonstrated almost constantly improving live birth rates following fresh non-donor (fnd) in vitro fertilization (IVF) cycles. Almost unnoticed by profession and public, by 2016 they, however, reached lows not seen since 1996–1997. We here attempted to understand underlying causes for this decline. This study used publicly available IVF outcome data, reported by the CDC annually under Congressional mandate, involving over 90% of U.S. IVF centers and over 95% of U.S. IVF cycles. Years 2005, 2010, 2015 and 2016 served as index years, representing respectively, 27,047, 30,425, 21,771 and 19,137 live births in fnd IVF cycles. Concomitantly, the study associated timelines for introduction of new add-ons to IVF practice with changes in outcomes of fnd IVF cycles. Median female age remained at 36.0 years during the study period and center participation was surprisingly stable, thereby confirming reasonable phenotype stability. Main outcome measures were associations of specific IVF practice changes with declines in live IVF birth rates. Time associations were observed with increased utilization of “all-freeze” cycles (embryo banking), mild ovarian stimulation protocols, preimplantation genetic testing for aneuploidy (PGT-A) and increasing utilization of elective single embryo transfer (eSET). Among all add-ons, PGT-A, likely, affected fndIVF most profoundly. Though associations cannot denote causation, they can be hypothesis-generating. Here presented time-associations are compelling, though some of observed pregnancy and live birth loss may have been compensated by increases in frozen-thawed cycles and consequential pregnancies and live births not shown here. Pregnancies in frozen-thawed cycles, however, represent additional treatment cycles, time delays and additional costs. IVF live birth rates not seen since 1996–1997, and a likely continuous downward trend in U.S. IVF outcomes, therefore, mandate a reversal of current outcome trends, whatever ultimately the causes.

A first report suggesting declines in U.S. live birth rates in fndIVF referred to years 2010–2014 [1]. Even steeper declines followed, however, in 2015–2016, reducing rates to levels not seen since 1996–1997 (Fig. 1) [2]. These findings attracted surprisingly little professional as well as media attention but have to be considered astonishing, considering how important pregnancy success is to infertility patients undergoing IVF [3] and considering that quality control processes in medicine usually strive for outcome improvements rather than declines. Declining IVF success is not only an alarming quality parameter but also suggests decreasing cost-effectiveness of IVF since declining live birth rates are usually compensated by increasing IVF cycle starts. Japan, in this sense, is a good example: As national live birth rates following fresh non-donor (fnd) IVF cycles plummeted by two-thirds, the country tripled IVF cycles initiations [1, 2].

**Fig. 1:**
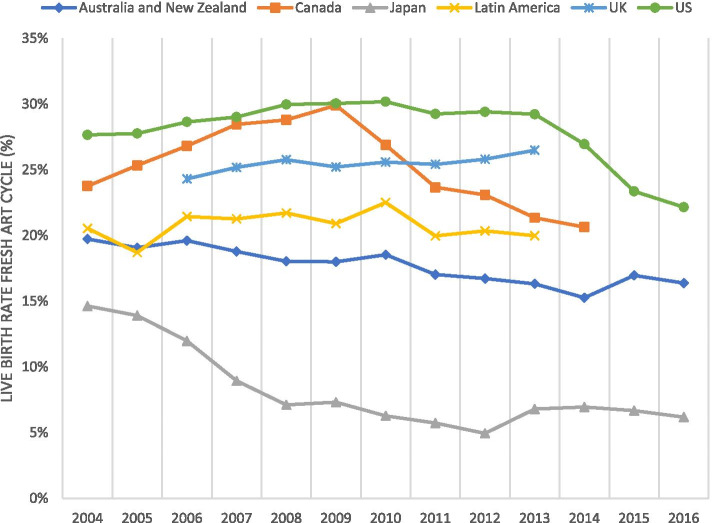
The figure demonstrates that autologous non-donor live birth rates in most regions of the world stagnated or fell during the study period. The most obvious decline occurred in Japan loosing approximately two-third of live births. Canada also demonstrated a significant drop, while Australia and New Zealand gradually lost approximately 25% of live births [Modified with permission from Kushnir et al.,2017 (1) and Gleicher et al., 2019 (2)]. Data are derived from reports from Australian & New Zealand Assisted Reproduction Database (ANZARD) (https://npesu.unsw.edu.au/..), *Canada Fertility & Andrology Society* Annual Reports (CARTR) (https://cfas.ca/cartr-annual-reports/) *Japan Society of Obstetrics & Gynecology* (*JSOG*, http://www.jsog.or.jp/modules/en/index.php?content_ id = 1); Latin American Network of Assisted Reproduction (REDLARA, www. redlara.com), *Human Fertilisation and Embryology Authority (HFEA)* for United Kingdom (www.hfea.gov.uk/fertility-clinics-success-rates.html), and the *CDC* for the U.S. (www.cdc.gov/art/artdata/)

Since in the U.S. IVF live birth rates until 2010 mostly steadily improved, the recent profound decline, representing almost a third of the national birth rate in fndIVF cycles, was unexpected. Similar declines have, however, during the same time interval also been observed in most other parts of the world (Fig. 1) [2].

Concomitant declines in fndIVF cycle outcomes in different geographic regions of the world, and involving varying genetic backgrounds of patient populations, suggest common underlying causes. In considering such causes, only newly introduce worldwide treatment changes to IVF can explain such parallel declines in IVF live birth rates all over the world. When such changes were introduced in different parts of the world is, moreover, well known and reasonably well documented in worldwide outcome reporting [1, 2]. Tracing these treatment changes in their respective timing to changes in fndIVF live birth rates, we previously pointed out potential causal relationships on a worldwide scale [2]. We have also noted that declines in fndIVF live births may, at least in part have been compensated by worldwide practice changes, like increasingly popular embryo banking, followed by delayed thaw-cycles. One, therefore, could argue that such frozen-thawed cycles must be considered in cumulative pregnancy and live birth rates. Though to a degree a valid argument, one then, however, must also consider the fact that a subsequent thaw cycle represents an additional treatment cycle, delays potential pregnancy and live birth, adds to already exorbitant (at least in the U.S.) IVF costs and, simply because basic logic leads to the conclusion that some cryopreserved embryos do not survive freezing and thawing, one must also acknowledge that cryopreservation simply must reduce pregnancy and live birth chances. We, therefore, here expand on prior worldwide observations with more detailed outcome observations in the U.S.

Details the CDC provides about U.S. IVF cycle outcomes are unique and have the potential of offering additional insights. This manuscript, therefore, builds on our previously published worldwide analysis [2] by extracting data from a steady longitudinal data set of U.S. IVF outcomes published by the CDC between 2005–2016. As will be demonstrated, these data significantly further strengthen the hypothesis that specific add-ons to IVF, introduced into routine worldwide IVF practice between 2010 and 2016, are likely causally related to steep declines in U.S and other fndIVF live birth rates around the world.

## Methods

### Participants

Annual summary reports of the National Assisted Reproductive Technology Surveillance System (ASR) have been published in mostly identical format by the *Center for Disease Control and Prevention (CDC)* since 2005 and are available at https://www.cdc.gov/art/reports/archive.html. We here reviewed ASRs between 2005–2016, with index years being 2005, 2010, 2015 and 2016. Reported data were extracted from the front-materials of the NASS, representing respectively, in 2005, 27,047; in 2010 30,425; in 2015, 21,771; and in 2016, 19,137 fnd live births in IVF cycles. They represent cumulative data from over 500 U.S. IVF centers, representing over 90% of U.S. IVF centers and over 95% of U.S. IVF cycles.

### Aggregate data

Since these are anonymous aggregate data, it is impossible to make statistical adjustments for crucial variables that may have changed over time, with female age, likely, being the most important one [4]. It, therefore, was reassuring that, despite reports of age of female infertility patients steadily increasing, median age of patients undergoing IVF cycles in the U.S. during the study period remained constant at 36 years. As will be discussed further in more detail below, this is, likely, explained by an overwhelming majority of IVF centers in the U.S. referring women above age 42 into third-party egg donation cycles. Increasing numbers of older women initiating infertility treatments, therefore, does not, as one might expect, automatically increases median ages in fnd IVF cycles and patients undergoing fnfIVF cycles remain steady in median age. Phenotypical stability of the study population is also suggested by stable center numbers reporting to the CDC, and with mostly only smaller IVF centers with relatively small IVF cycle numbers not following the Congressional reporting mandate. There is also no specific reason to believe that percentages of repeat cycles changed significantly, though one must acknowledge that lower pregnancy success in later years may, as Japanese data suggest [1, 2], have increased the number of repeat cycles. Increasing numbers of repeat cycles tend to reduce pregnancy rates. This study, therefore, cannot rule out a small additional contribution to falling live birth rates from declining age-dependent pregnancy and live birth rates, themselves.

### Time associations with introduction of new add-ons to IVF

IVF outcome changes in ASR data were then in timeline compared with reported introductions and changes in utilization of new add-ons to IVF in the U.S. Such associations do not indicate causation but can be strongly hypothesis-generating if timelines coincide in introduction of an add-on, if effects on IVF cycle outcomes increase with increasing utilization of add-ons and if similar observations are made around the world, involving different racial and ethnic populations. In addition, phenotypically consistent and stable patient populations, like here discussed U.S. IVF population, in longitudinal observations allow for further inferences. Beyond criticism about unvalidated introductions of new add-on treatments made by others [5], here offered commentaries on observed associations are not meant as criticisms of specific add-ons per-se, but as inducement for the conduct of overdue studies to define the clinical utility of these new add-ons to IVF.

### Statistical analysis

Data are presented in 5-year intervals, with 2005, 2010, 2015 and 2016 as index years. For graphic reasons, figures, however, are also filled in for the years in-between. In 2016, 463 out of 502 (92.2%) U.S. IVF centers reported. Statistical analyses to compare years were performed by our center’s statistician (see acknowledgment), using Chi-square and with a *P* < 0.05 considered statistically significant. All statistical analyses were preformed using SAS version 9.4.

## Results

### Changes in IVF cycle type distributions

**Fig. 2:**
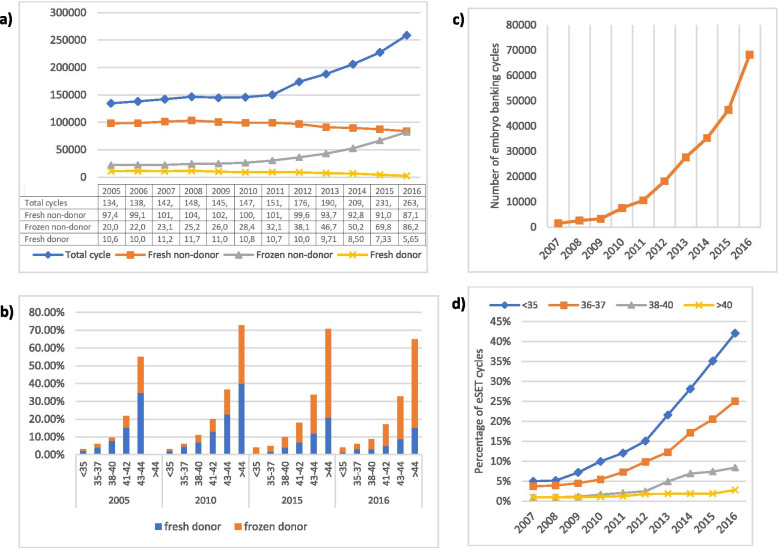
**(a)** The biggest contribution to increases in total cycle numbers came from frnd-cycles, likely, the single most important practice change during the study period. **(b)** This figure demonstrates progressive increases in donor egg cycles with advancing female age, starting at age 38 but accelerating above age 40 and especially after age 42 years. The figure also demonstrates the recent dramatic switch from fd- to frd-cycles. **(c)** Utilization of embryo banking significantly increased over the study period, with the year 2010 representing the beginning of a significant uptick. **(d)** This figure demonstrates the significant increase eSET utilization in women of all ages, with greatest increases in youngest patients but even women above age 40 experiences increases. Source for all figures: www.cdc.gov/art/artdata/

All-inclusive, the U.S. performed in 2005, the first year of this study, 134,618 assisted reproductive technology cycles (Fig. 2a). This included fnd -cycles, frozen non-donor (frnd)-, fresh donor (fd)-, and frozen donor (frd)- cycles.

By 2010, 147,264 cycles were performed (+ 9.39%); yet, fnd-cycles declined for the first time from 72.4% to 68.5% of all cycle activity (*P* < 0.0001), while frnd-cycles increased from 15.3% to 19.3% (*P* < 0.0001), fd-cycles decreased from 7.9% to 7.4%, (*P* < 0.0001) and frd-cycles increased from 4.1% to 4.9% of all cycles (*P* < 0.0001). The year, thus, demonstrated significant first declines of fresh in favor of increasing frnd-cycles and a similar trend from fd- to frd-cycles, while, overall, d-cycles increased only marginally from 12.0 to 12.3% of all cycles.

By 2015, these trends further strengthened, with fnd-cycles demonstrating significantly further decreased (68,5% to 39.3%, *P* < 0.0001), while frnd-cycles substantially increased (19.3 to 30.1%, *p* < 0.0001). Remarkably, by 2016, fnd- and frnd-cycles, indeed, had pulled even at 32.7%, each. Concomitantly, “all-freeze” (EB) cycles increased from 19.7 to 25.0% of all cycles (*P* < 0.0001). Further changes in donor egg cycles are discussed below (Fig. 2b).

These data, thus, demonstrate a highly significant and substantial switch from fresh to frozen-thawed IVF cycles. One, therefore, must assume that such substantial changes in practice must follow one or more purposeful intents, discoverable in the medical literature. Yet, surprisingly, we were unable to discover a convincing rational for such a drastic practice change, leaving unanswered why these practice changes occurred. Here following observations may, however, offer some answers.

### “All-freeze” cycles: Elective Embryo Freezing (EEF) and Embryo Banking (EB)

Both IVF cycle types cryopreserve all embryos an IVF cycle yields but do so with varying clinical intent: EEFs are frequently utilized to improve IVF outcomes when the endometrium is believed out of sync with embryo stage because of ovarian hyperstimulation [6]. EB, on the other hand, is mostly an embryo-accumulation strategy, often utilized in poor prognosis patients in association with mild ovarian stimulation. Though in registries this term is reserved for embryos that are frozen for at least one year, we here use it independent of length of cryopreservation. Fortunately an increasingly rarely encountered complication, attempts at avoiding the ovarian hyperstimulation syndrome (OHSS), may on rare occasions also lead to all-freeze cycles.

Clinical utilization of both practice patterns has significantly increased in recent years (Fig. 2c). Above noted U.S. data demonstrated that over the study period of 16 years, cycles using fresh embryos declined from 72.4% to 32.7% (54.8%; *P* < 0.0001), while frozen cycles increased from 15.3% to 32.7% of all IVF cycles (113.8%, *P* < 0.0001). All-freeze cycles because of hyperstimulation are, of course, included in these numbers and cannot separated out in CDC data. They, however, always represented only a small minority of cycles and, moreover, have significantly declined over the last decade due to improvements in preventive measures taken during IVF stimulation and in triggering ovulation.

The literature suggests that outcome benefits from EEF are only achieved in true hyper-responders at risk for OHSS and, possibly, with preimplantation genetic testing for aneuploidy (PGT-A) [7]. The latter is, in itself, a highly controversial practice. Except in selected good-prognosis patients, “all-freeze” strategies, indeed, appear futile and maybe even harmful. This was, likely, best demonstrated in two recent large prospectively randomized multicenter studies in genetically homogenous patient populations (Han Chinese) which produced seemingly contradictory results [8, 9]. One claimed outcome advantage for frozen over fresh elective single blastocyst-stage embryo transfers (eSETs) (8); the second, however, was unable to demonstrate such outcome differences [9]. There was only one crucial difference: The first study transferred embryos at blastocyst-stage, while the second study replaced embryos at cleavage-stage. Only relatively good-prognosis patients, however, produce blastocyst-stage embryos. This first study, therefore, favorably selected patients, while by transferring embryos already on day-3 after fertilization, the second study did not. Both randomized studies, thus, came to opposing conclusions only because patient populations differed in subtle ways. Summarizing he literature, one, therefore, must conclude that in unselected patient populations an “all-freeze” strategy does not appear to improve IVF outcomes.

A study’s conclusions cannot be automatically applied beyond populations in which a study is performed. Inappropriate extrapolations have, however, over the last decade repetitively been the reasons for IVF practice changes. Specifically, studies in highly selected good-prognosis patients have repeatedly been generalized to poorer-prognosis populations in such important areas of IVF as, routine extended culture to blastocyst stage, elective single embryo transfer (eSET), PGT-A and, as here noted, “all-freeze” cycles. Outcomes in IVF, however, greatly vary between better and poorer prognosis patients. What, therefore, may constitute a winning strategy in good prognosis patients may be a losing strategy in poorer-prognosis patients.

Above noted two Chinese studies [8, 9], are not alone in refuting the EEF concept [10, 11]. It, therefore, is still difficult to understand on what basis in recent years so many U.S. IVF cycles have become “all-freeze” cycles (Fig. 2c).

Kushnir et al. may have offered some insights: They demonstrated how “all-freeze” cycles potentially biased IVF outcome reporting by allowing under current CDC guidelines for IVF cycle outcome reporting selected embryo transfer deferrals into future thaw cycles. Doing this with poorer- prognosis patients, removed these cycles from reporting obligations to the CDC and, therefore, at least hypothetically, raises a center’s pregnancy and live birth rates for remaining better-prognosis patients. The authors also reported that, counterintuitively, because of in principle declining success of cryopreservation with advancing female age, the 10 U.S. centers with highest EEF/EB utilization of “all-freeze” cycles were, indeed, preferably freezing embryos in poorer-prognosis patients [12, 13]. After adjusting IVF outcomes reported to the *CDC* by these 10 centers from reference “per transfer” to “per cycle start” (intent to treat), Kushnir et al. demonstrated that their live birth rates, actually, fell from exceptional high to below median for all other approximately 500 reporting U.S. IVF centers [13].

The reason for this outcome discrepancy was, of course, again selection of good-prognosis and unselecting of poorer prognosis patients, thus establishing a repetitive theme for this study: Add-ons and related IVF practice changes, alone, are not the only culprit for declining live birth rates in fndIVF cycles. Misleading statistical outcome reporting is an almost equally important contributing factor and must be curtailed. An ultimate solution, however, requires even more: Consistent outcome reporting must be able to follow practice changes until a fresh cycle’s cumulative pregnancy/live birth potential has been exhausted. Only such cumulative cycle outcomes will, ultimately, allow for unbiased outcome reporting in IVF [14].

### The kato protocol

Developed in Japan [15], this protocol quickly became the country’s dominant IVF protocol. As a consequence, and with no other reason detectable, Japan, subsequently over eight years (2004–2012), lost by intent to treat (reference cycle start) almost two-thirds of live births following fndIVF cycles, with the rate, ultimately, declining into the 5–6% range (Fig. 1) [1]. To maintain overall live birth numbers, the country, however, tripled in parallel its cycle starts [1, 2], raising obvious questions about cost-effectiveness in addition to obvious quality of care issues.

The protocol is defined by mild ovarian stimulation, mandated EECTB and eSET [15]. Once again, blatantly incorrect statistical outcome reporting [15, 16] contributed to its popularity. Neither transparent criticism [17] nor acknowledgment of significantly lower pregnancy chances by proponents of the protocol [18] did stop the protocol’s utilization. It does eliminate OHSS risk and, since associated with eSET, minimizes twin pregnancies. No support, however, exists for the claim of better egg/embryo quality with milder ovarian stimulation [19]. Lower gonadotropin consumption is, likely, a fact but, as also claimed, lower treatment costs with great likelihood are only a myth, considering the need for additional cycles in compensation for lower live birth rates [20]. Like other practice changes to IVF, the protocol’s worldwide utilization is, therefore, difficult to understand.

Though not as popular as in Japan, Kato or similar protocols, have also found a following in the U.S. Because of lower live birth rates than standard stimulations, they, therefore, unquestionably also contribute in the U.S. to falling national fndIVF outcomes, though, obviously, not to the same degree as in Japan. How the Kato protocol was presented to public and professional community, likely, played an important role in its ascendance and popularity. Marketed as: “more natural” (because of mild stimulation), “more economical” (because of less medication costs and less need to monitor patients), “less costly” (since cycle costs are lower in comparison to traditional ovarian stimulations) and, therefore, as “more cost-effective,” facilitated successful, often direct-to-consumer, marketing of the protocol as “more patient-friendly.” The protocol’s individual components are discussed next since they, also individually in association with other ovarian stimulations, contribute to declining IVF pregnancy and live birth rates.

### Extended Embryo Culture to Blastocyst stage (EECTB) and Elective Single Embryo transfer (eSET)

First proposed by Gardner et al. as an embryo selection method facilitating eSET, the purpose of EECTB was to minimize time to pregnancy and, in association with eSET, reduce twinning [21]. Especially when it comes to reductions in twin pregnancies, this treatment strategy has been successful. Embryo selection, will, however, only improve outcomes if more embryos are available than one is willing to replace.

Revisiting previously noted theme of patient selection in important studies that have changed IVF practice, Gardner et al. included in their initial study exclusively only good-prognosis patients who produced large egg and embryo yields. As an embryo selection (and not an embryo replacement) method, follow-up studies in unselected patient populations, therefore, unsurprisingly were uniformly unable to duplicate the results of Gardner et al. [21]. It is now widely accepted that, to draw even in live birth chance with a fresh two-embryo fresh transfer, eSET after EECTB requires a second frozen-thawed embryo transfer cycle [3]. Otherwise, an eSET will be deficient in pregnancy and live birth rates in comparison to a two-embryo transfer.

The need for additional IVF cycles because of recommended practice changes in IVF, has become an important new issue for discussion in determining and/or validating proposed changes to IVF practice because every loss in pregnancy chance can, of course, be compensated for by initiating more IVF cycles, whether fresh or frozen-thawed. We previously noted that Japan compensated for a two-third national loss in live birth rates in fresh IVF cycles by tripling their IVF cycle starts [1]. Within such a context, it is also important to reemphasize that in this study demonstrated declines in live birth rates in fresh IVF cycles partially may be compensated by deferred transfers in frozen-thawed cycles not included in here reported live birth rates. But these additional pregnancies and live birth rates always come at significant additional economic and emotional price, caused by delay in treatment, additional cycle starts and, ultimately, higher cost.

We here also witness yet another typical clinical circumstance where information developed in good-prognosis patients has been inappropriately generalized, as most IVF centers now consider EECTB an appropriate standard method of embryo culture for all IVF patients. The potential damage to live birth rates in fndIVF cycles in such centers, then, becomes obvious because embryos of poorer prognosis patients, often, do not reach blastocyst stage in extended culture. Such patients, therefore, are deprived of all pregnancy chances, as small as those may be with earlier cleavage-stage embryo transfers.

Two Cochrane metanalyses offer further information [22, 23]. They suggested that cumulative pregnancies and live births (using a cycle’s complete embryo cohort) do not differ between cleavage stage (day-3) and blastocyst stage (days-5/6) transfers. This finding, however, has further connotations because it suggests that, in absence of outcome differences between cleavage- and blastocyst-stage transfers, day-3 transfers in intermediate- and poor-prognosis patients must in some ways compensate for outcome advantages of good-prognosis patients at blastocyst stage, which Gardner et al. convincingly established. [21]. Selected embryos that do not survive EECTB, if transferred at cleavage stage, must, therefore, still result in pregnancies. This was further confirmed in a recently published study of women who at cleavage stage had only one embryo available for transfer. They then were randomized to cleavage stage transfer of that embryo or to extended culture of that embryo to blastocyst stage and transfer on days 5/6 after fertilization. Outcomes to highly significant degrees favored cleavage-stage embryo transfer, thereby reaffirming that some cleavage stage embryos that do not survive extended culture, if transferred earlier, still, will result in normal pregnancies [24].

At least in poorer-prognosis patients, EECTB, therefore, causes adverse outcome effects on live birth chances in fndIVF cycles, further aggravated by eSET. The impact of these adverse effects, of course, grows with increasing utilization and U.S. as well as international data clearly demonstrate rapidly increasing utilization of EECTB and eSET. Increasing adverse effects on outcomes, therefore, appear very likely. Indiscriminate utilization of eSET has been common practice in Europe even before 2009–2010 [25]. In the U.S., utilization, however, increased especially rapidly after 2010 and even included women above age 40 (referenced in Fig. 2d) [25]. An adverse effect on fndIVF cycles can, therefore, be presumed.

We noted earlier that eSET produces lower birth rates than two-embryo transfers [26, 27]; but eSET is also convincingly associated with declining multiple pregnancies (Table 1). Losses in birthrates with eSET can be compensated for by an additional thaw cycle [26, 27]. Those compensatory births, however, do not appear in the *CDC’s* ASRs of fndIVF cycles. Like EECTB, growing eSET utilization, thus, as noted before, potentially reduces fnd-live birth rates,

**Table 1:** A longitudinal view of single and multiple fetus clinical pregnancy rates, 2005–2016

Study year	2005	2010	2015	2016	*p*-value
Clinical pregnancies (%)^a^	34.5	36.8	29.3	25.4	< 0.0001
Singleton (%)	20.5	23.1	20.4	19.8	< 0.0001
Multiples (%)	11.2	11.5	6.9	5.6	< 0.0001
No pregnancy (%)	65.4	62.4	70.2	72.2	< 0.0001

This table demonstrates that multiple fetus clinical pregnancy rates after 2010 declined more than singleton pregnancies, suggesting a successful national U.S. effort to reduce multiple pregnancies and deliveries

^a^With reference point cycle start

As simple time associations, correctly, remain suspect in being presumed to establish causation, it is, nevertheless, interesting to note that national data from the U.S., Japan, Canada and Australia/ New Zealand lead to similar conclusions [1, 2], and worldwide consensus that eSETs reduces multiple births was, basically, only reached based on similar association studies [28, 29]. Further studies to clarify the respective contributions of EECTB and eSET to declining fndIVF live birth rates, therefore, appear indicated [30, 31].

### Preimplantation Genetic Testing for Aneuploidy (PGT-A)

Ten years following a first pronouncement by the *American Society for Reproductive Medicine* (*ASRM)*, the Practice Committees of *ASRM* and *SART* again concluded that PGT-A lacks clinical efficacy [32]. Due to high rates of false-positive diagnoses [33], PGT-A actually reduces conception chances in at least selected patient groups [34]. Despite deliveries of hundreds of healthy offspring following transfers of embryos, by PGT-A originally diagnosed as chromosomal abnormal [35], the test, still, enjoys popularity. Besides of the high false-positive rate of the test and, therefore, because of non-use of many perfectly normal embryos in IVF cycles, adverse effects on IVF cycle outcomes are certain. Yet, like the Kato protocol [15], PGT-A, in addition, potentially also exerts adverse secondary effects on fndIVF outcomes through mandated EECTB and “all-freeze” cycles, required by how the latest version of PGT-A is practiced. Especially in poorer prognosis patients with small embryo numbers, declines in fndIVF outcomes are, therefore, multifactorial and cumulative.

For all of these reasons and, considering above noted ASRM/SART position on PGT-A [32] as well as potentially negative outcome effects on at least select patient populations, continued utilization of PGT-A in general infertility populations and in routine IVF cycles has been increasingly questioned. Two recent editorials [36, 37], following publication of the so-called STAR study, which again failed to demonstrate outcome benefits from PGT-A [38], suggested serious reconsiderations in how PG-A should be utilized in association with IVF. Strong financial incentives for continued utilization of PGT-A, shared by IVF centers and PGT-A laboratories, make a quick reversal of current practice, however, somewhat unlikely [39].

Because PGT-A requires routine EECTB and “all-freeze” cycles, the procedure has affected IVF practice beyond just performance of embryo biopsy and laboratory testing for chromosomal abnormalities. Unquestionable, PGT-A has also in general IVF practice greatly contributed to increasing routine utilization of EECTB as well as of “all-freeze” cycles. PGT-A may, therefore, be the most consequential add-on to IVF practice over the last decade, contributing significantly to observed declines in live birth rates in fndIVF cycles. In addition, PGT-A also again demonstrates how dangerous extrapolation of outcome data from highly selected patients to general patient populations can be, with women with small embryo numbers being more negatively affected than good-prognosis patients with larger embryo populations.

The following important time-points in the evolution of PGT-A document well the likely association between utilization of PGT-A and declining live birth rates in IVF: Already in 2007, Mastenbroek et al. demonstrated that, what then often was called PGS 1.0, was ineffective in improving IVF cycle outcomes. The authors, moreover, also were the first to demonstrate that the procedure in older women, actually, reduced pregnancy chances [39]. Following another publication that made the same point after reanalyzing a small prospectively randomized Belgian study [40], the *ASRM* for the first time concluded in 2008 that, what then was called PGS, lacked beneficial outcome effects [41]. When around 2010, PGS 2.0 was introduced, clinical pregnancy and live birth rates in IVF practice were at their peak (Fig. 1). PGS 2.0 moved embryo biopsy from cleavage- to blastocyst-stage [42], thus mandating EECTB and” all-freeze” cycles in association with PGT-A. As discussed in further detail below, the year 2010, therefore, became a crucially important turning point in the worldwide clinical practice of IVF.

### Changes in donor egg cycles (dIVF) with advancing female age

We previously noted why third-party egg donation is highly relevant for fndIVF cycle outcomes: Since an overwhelming majority of IVF centers after age 42 no longer offer women IVF with use of their own eggs (https://www.cdc.gov/art/artdata/index.html), remaining U.S. patients in fndIVF cycles over here presented study period have maintained a median age of 36 years, even though the average age of women seeking out fertility treatments in general has been steadily increasing. Widely considered a center of last resort, and offering autologous oocyte use into advanced ages, our own center’s median patient age, for example, has risen to 43 years between 2017–2019 (personal communication by the authors).

All of this is highly relevant to outcome assessments of fndIVF cycles because the more aggressively an IVF center utilizes third party egg donation, the more favorably selected will the remaining patient pool at any given IVF center be that is, still, offered use of autologous oocytes. dIVF cycles, thus, remove poor prognosis patients from patient pools, leaving behind a patient population with better prognoses and, therefore, better pregnancy and live birth rates following fndIVF cycles. Whether such considerations come into play in treatment selections at IVF centers is not subject of this communication. Relevance of this discussion is, however, apparent in the indisputable observation that, for these reasons, what has happened to dIVF cycles over here presented study period matters. Moreover, increases in utilization of donor eggs should, therefore, if anything, improve fndIVF cycle outcomes. A decline in dIVF cycles, in contrast, should lead to an expectation of declining pregnancy and live birth rates, as more poor prognosis patients use their own eggs.

As Fig. 2b demonstrates, dIVF cycles peaked especially in oldest women around 2010 and have since then been relative stable. Changes in utilization of dIVF cycles, therefore, likely have not significantly affected fndIVF cycle outcomes. As expected, throughout the study period, dIVF cycles increased with advancing age, from 3–4% of all cycles under age 35, to 5–6% between 35–37, 9–11% at ages 38–40, 17–22% at 41–42 years, and 33–37% at ages 43–44. Above 44 years, they in 2010 represented as much as 73%, but by 2015, only 71%, and by 2016, only 65% of cycles. After 2010, slightly more women, therefore, underwent fnd-IVF. The potential impact from this observation between 2010–2016 would actually be that of a minor reverse patient selection, with women in fnd-IVF cycles becoming prognostically somewhat more unfavorable. Due to stable patient ages, this effect can, however, not have been substantial but must, nevertheless, be considered. Starting again in 2010, switching aggressively, especially above age 42, from fresh to frozen donor eggs, sped-up utilization of frdIVF cycles (Fig. 2b). Overall declining dIVF cycles, however, suggest that patients have been getting better chances to use their own autologous eggs, a to be welcomed development [43].

The radical shift from fd to frd cycles after 2010 has not been reported before in the literature. It follows establishment of several frozen egg banks in recent years. Oocyte banking offers a number of advantages over use of fresh eggs, such as simplification of treatment processes, easier transportation of gametes and embryos and greater donor choices [44]. It, however, also has downsides: The most important appears to be a significant outcome advantage of fresh over frozen donor eggs. When first reported [44], such differences were disputed, and better outcomes were predicted with improving experience in donor egg banking [45]. With further observation, the discrepancy between fresh and frozen donor eggs, however, expanded to an approximately 10% difference in pregnancy rates [46].

### Why outcome analyses with reference cycle start must be the rule

Misdirection of reporting of clinical outcome data has here repeatedly been identified as a principle cause for misdirections in clinical IVF practice over the last decade. This has also been the case when IVF cycle outcomes are widely reported with reference point embryo transfer rather than reference point cycle start (intent to treat). Here is a relevant example (Table 2): Between 2005–2016 fnd-cycles declined by 37.1%, while live birth rates (with reference cycle start) lost absolutely 8.0% and relatively 26.5% after peaking in 2010. If calculated with reference embryo transfer, IVF cycle outcomes, however, remained stable (36.3% to 36.8%). Moreover, rates per embryo transfer were obviously higher than per cycle start and, at + 6.7% and + 6.4%, respectively, remained stable between 2005 and 2010,—only to accelerate to + 12.8 and + 14.1% by years 2015 and 2016 (Table 2). Outcome reporting with reference point embryo transfer, therefore, distorts IVF practice and, especially after 2010, has increasingly obfuscated declining U.S. IVF outcomes.

**Table 2:** Differences in reporting of live birth rates depending on reference point, 2005–2016

Study year	2005	2010	2015	2016	*p*-value
fnd-cycles n	27,947	30,425	21,771	19,137	
Live birth rates/cycle- start (%)	27.8	30.2	23.9	22.2	< 0.0001
Live birth rates/embryo transfer (%)	34.5	36.8	36.7	36.3	< 0.0001
Gain in absolute percentage points	+ 6.7	+ 6.4	+ 12.8	+ 14.1	< 0.0001

This table demonstrates the gain in absolute percentage points for IVF centers when reporting live births with reference point embryo transfer rather than cycle start. The gain more than doubled after 2010, reflective of more “all-freeze” cycles and other IVF practice changes after 2010

## Discussion

With national U.S. live birth rates per cycle start in fndIVF cycles precipitously declining after 2010, and especially after 2013, this manuscript attempted to demonstrate changes in IVF practice which during the study period of 2005–2016 may have been responsible for these changes. Since, affecting patients of different races and ethnic backgrounds at identical times, and since similar declines have also been reported in other regions of the world [1, 2], it appears reasonable to conclude that worldwide practice changes, introduced to IVF practice influenced U.S. and worldwide practice at similar time points. Observations made, based on almost complete national reporting by all U.S. IVF centers, therefore, likely may, at least partially, also apply to other regions of the world.

A word of caution in interpreting here presented data is, however, indicated: As over the study period increasing numbers of embryos were cryopreserved rather than freshly transferred, they, of course, represent additional potential pregnancy and live birth chances in the future. One, therefore, at least hypothetically, may argue that this potential should be added to live birth rates achieved and here reported over the study period. At least embryos frozen with the intent of later transfer should be added but such data cannot be extracted from current U.S. reporting systems. Though this caveat unquestionably represents a weakness of here reported analysis, it can be countered by previously noted observations that, as freezing rates increased over the study period, U.S. IVF centers preferentially froze embryos from poor prognosis patients [12, 13], which, of course, can be expected to produce only relatively low pregnancy chances after thawing. It, therefore, appears unlikely that, from a patient’s point of view, here presented consequences of add-ons to IVF would look much more positive, even if pregnancies and live births from frozen-thawed cycles were to be added.

Likely, the most important point to be made in this regard is, however, that add-ons were supposed to improve IVF outcomes; if they were to produce only similar results, what would be their purpose? To reemphasize, though any outcome deficit in IVF can be statistically compensated by performing more IVF cycles, this should not be accepted as a valid argument in supporting changes to IVF practice that reduce immediate pregnancies and live births following fndIVF cycles *unless* a practice change offers a compensatory benefit. Here is an example: As previously reviewed, eSET reduces pregnancy and live birth rates in comparison to 2-embryo transfers, requiring a compensatory frozen-thawed cycle to pull even. Nobody, therefore, can argue that, solely based on comparisons between eSET and 2-embryo transfer cycles, eSET should be the preferred clinical approach. The reason why proponents of eSET do, however, have a potential argument, is a parallel claim of a secondary benefit from eSET in reducing twin pregnancy rates, which they associate with increased outcome risks for mothers as well as offspring. Without this claim which, as also previously addressed, has remained controversial, eSET would, likely, not even be a point of discussion.

As Table 1 demonstrates, the year 2010 must be viewed as turning point because this was clearly the year when preceding constant improvements in IVF cycle outcomes in the U.S. (and often elsewhere) came to a screeching halt, initially plateaued, but within a few short years started to reverse. A closer examination of what transpired around the year 2010 in IVF practice, therefore, appears in place.

### What the year 2010 is telling us

**Fig. 3:**
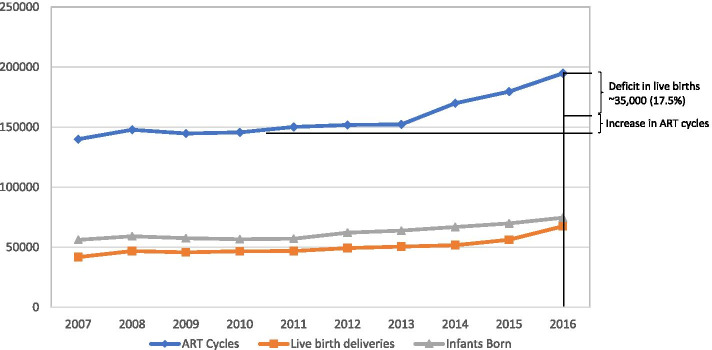
A comparison of total ART cycle starts with live births and infants born 2007–2016 This figure demonstrates the discrepancy between live births and ART cycle starts over the study period. As is very obvious cycle starts ran, more-less, in parallel between 2007–2010; but starting with 2011 increasingly diverged. By 2016, the deficit in live births reached ~ 35,000 (17.5% of cycle starts). The figure also shows a mild convergence between live births deliveries and infants born, again demonstrating a relative decline in multiple births. On a national level this decline, however, further exacerbates the number of “lost” live births for the country. This figure does not take into consideration that cumulatively frozen-thawed cycles may have compensated for some lost pregnancy and live births in fresh cycles, as in detail discussed in the text. Modified from www.cdc.gov/art/artdata/

Demonstrating peaks in live births rates, the year 2010 represented an important turning point for IVF outcomes in the U.S., Latin America and Australia/New Zealand and, likely, Canada, had Quebec province not entered into an eSET agreement with the local IVF physician community [47] that plummeted birth rates (Fig. 1) [48]. In the U.S., effects are best demonstrated when comparing cycle-starts with number of live births (Fig. 3). Starting in 2010, a narrowing between live birth and infant birth curves suggests a life birth deficit and declining multiple pregnancies. By 2016, cumulatively, ca. 35,000 live births were annually lost. Embryo banking (“all-freeze” cycles also accelerated around 2010. (Fig. 2c) and the precursor of PGT-A, PGS 2.0, started replacing its own precursor, PGS 1.0, thereby increasing utilization of EECTB and “all-freezes” [49]. According to a prominent fertility website, already a few years ago approximately 35% of U.S. IVF cycles utilized PGT-A (https://www.fertilityiq.com/topics/pgs-and-ccs-genetic-testing/ criticisms-of-pgs). As even dIVF now utilize the procedure [50], the numbers as of 2020 must be above 50%.

Without apparent scientific rational, the year 2010 can be considered as starting point of radically new practice patterns in IVF, often not only characterized by absence of supportive evidence for newly introduced treatments and/or of prior validation studies, but also absence of rational support for such treatments. In addition, one must also acknowledge that an apparently compromised peer review process permitted biased outcome reporting, based on, at times, statistically incorrect data analyses.

Considering the rather astonishing success IVF has witnessed till 2010, here pointed out developments since 2010 are regretful, must be further investigated and, ultimately, reversed. Professional organizations and specialty journals in the field, moreover, must shoulder the responsibility of information dissemination that meets appropriate standards of evidence. Patients who seek out infertility treatments are usually highly motivated but also highly vulnerable to wishful thinking. They deserve better than treatment guidance that often is demonstrably incorrect.

## Conclusions

We in this manuscript, we believe convincingly, point out unfavorable IVF outcome trends in the U.S. between 2005 and 2016 which have received surprisingly little attention in the medical literature and also have not been communicated to the public, even though previously described in a study of worldwide trends [1]. Based on detailed U.S. registry data from reporting IVF centers, this study, however, associates these adverse outcome trends even closer to introduction of new IVF practices to IVF during this time periods, by others given the acronym “add-ons” [5].

Associations, of course, do not denote causation and are only hypothesis-generating. Here presented data, therefore, should not be understood as final evidence for here outlined losses in pregnancy and live birth rates being caused by these practice changes alone but, considering that similar changes have been observed worldwide in different regions and in timeline always similarly associated with introduction of the same “add-ons” to IVF practice in patient populations with varying genetic backgrounds, makes such associations highly probable. Considering furthermore that practically none of those new “add-ons” have been properly validated in their promised benefits to IVF practice [5], the IVF field must change direction in how it handles introduction of new practice patterns into IVF if further declines in the efficacy of IVF to lead to pregnancy and live birth are to be avoided.

## Data Availability

All data is contained within the manuscript.

## Abbreviations

CDC: Center for Disease Control and Prevention
ASRM: American Society for Reproductive Medicine
dIVF: Donor egg IVF
EB: Embryo banking
EECTB: Extended embryo culture to blastocyst
EEF: Elective embryo freezing
eSET: Elective single embryo transfer
fd: Fresh donor
fnd: Fresh non-donor
frd: Frozen donor
frnd: Frozen non-donor
IVF: In vitro fertilization
NASS: National Assisted Reproductive Technology Surveillance System
OHSS: Ovarian hyperstimulation syndrome
PGS: Preimplantation genetic screening
PGT-A: Preimplantation genetic testing for aneuploidy
